# Supplementary figures and images for: SweepCluster: A SNP clustering tool for detecting gene-specific sweeps in prokaryotes
Source: BMC Bioinformatics. 2022 Jan 6;23:19. doi: 10.1186/s12859-021-04533-6 (PMC8734265; doi:10.1186/s12859-021-04533-6)

Fig.S2

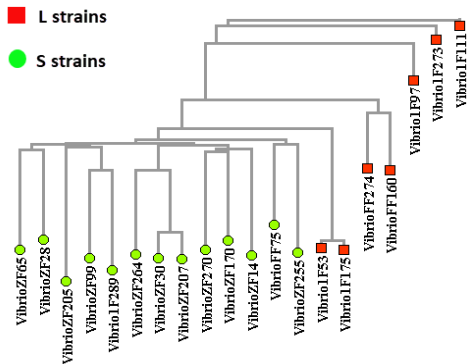

Supplement: Supplementary file 10 — Additional file 10: Fig. S2 Phylogenetic tree of the 20 strains of V. cyclitrophicus. The ecological partition of the strains is indicated in color for thirteen S strains and seven L strains. [file 12859_2021_4533_MOESM10_ESM.pdf]

Fig. S3

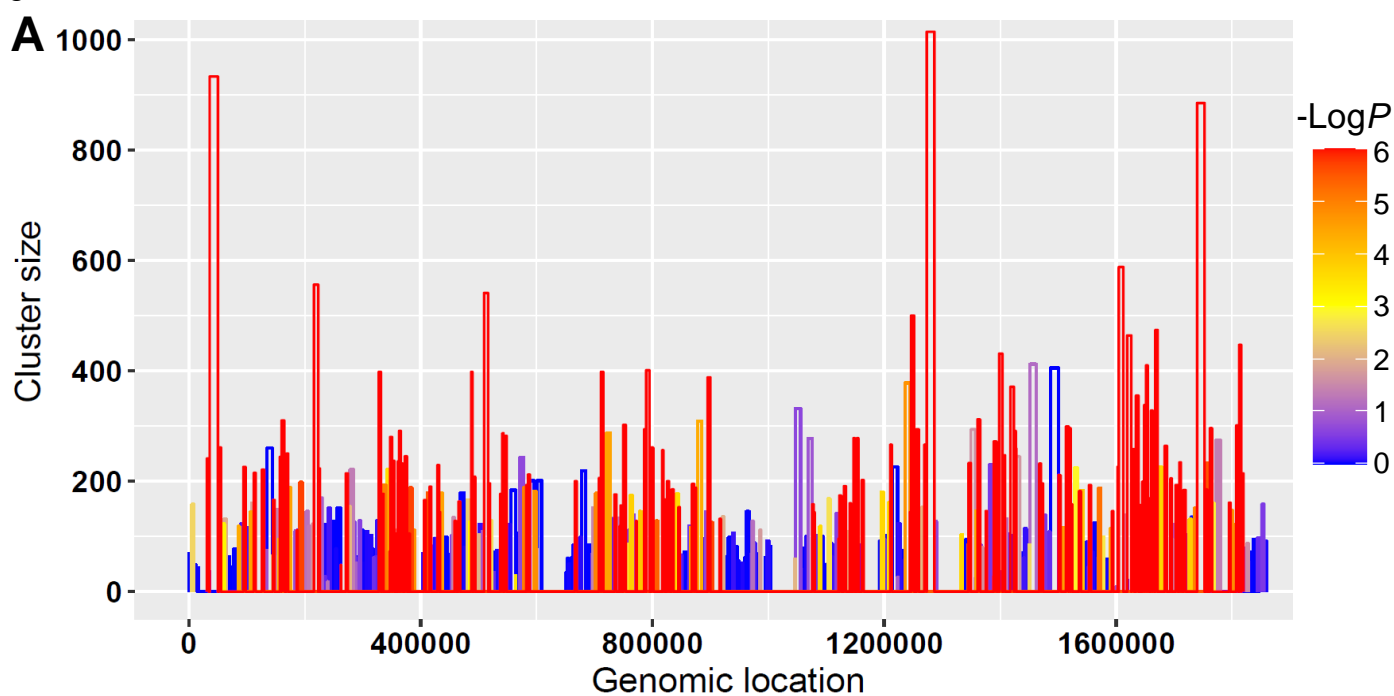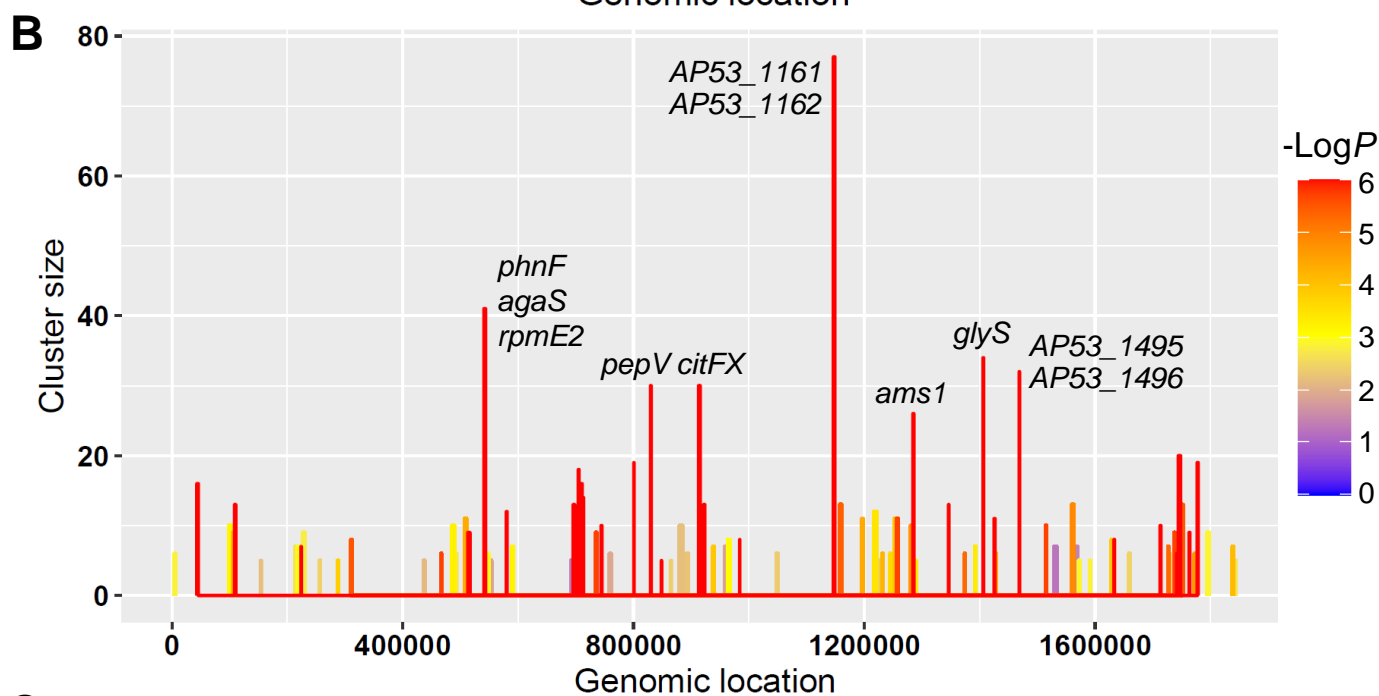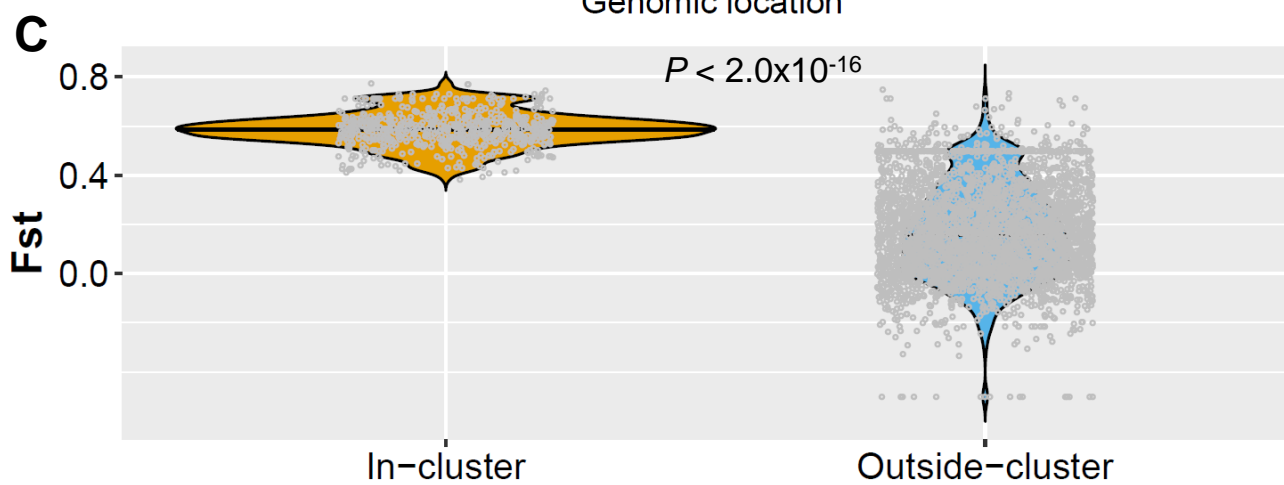

Supplement: Supplementary file 11 — Additional file 11: Fig. S3 SNP clusters with signatures of selective sweep identified by SweepCluster for S. pyogenes genotype datasets. (A) The clusters detected from all segregating SNPs in the core genome. (B) The clusters detected from 1,631 selected SNPs with phenotypic association (Chi-squared test p-value ≤ 0.001). The clusters are represented as colored bars with the bar height indicating the cluster size (the number of SNPs in the clusters) and the bar width indicating the spanning length. The significance of the clustering evaluated with -log10 (p-value) is indicated in gradient colors. The gene loci in the top clusters are shown. (C) Distribution comparison of population differentiation Fst between two groups of SNPs: (i) those falling into the 131 significant clusters involving 1201 SNPs and (ii) those outside of clusters among the whole-genome core SNPs. The distribution of Fst for the latter group was constructed using a random sampling of a quarter of the 67,540 core SNPs outside clusters and the calculation of Fst was based on a window size of 10. [file 12859_2021_4533_MOESM11_ESM.pdf]

Fig. S4

**A**

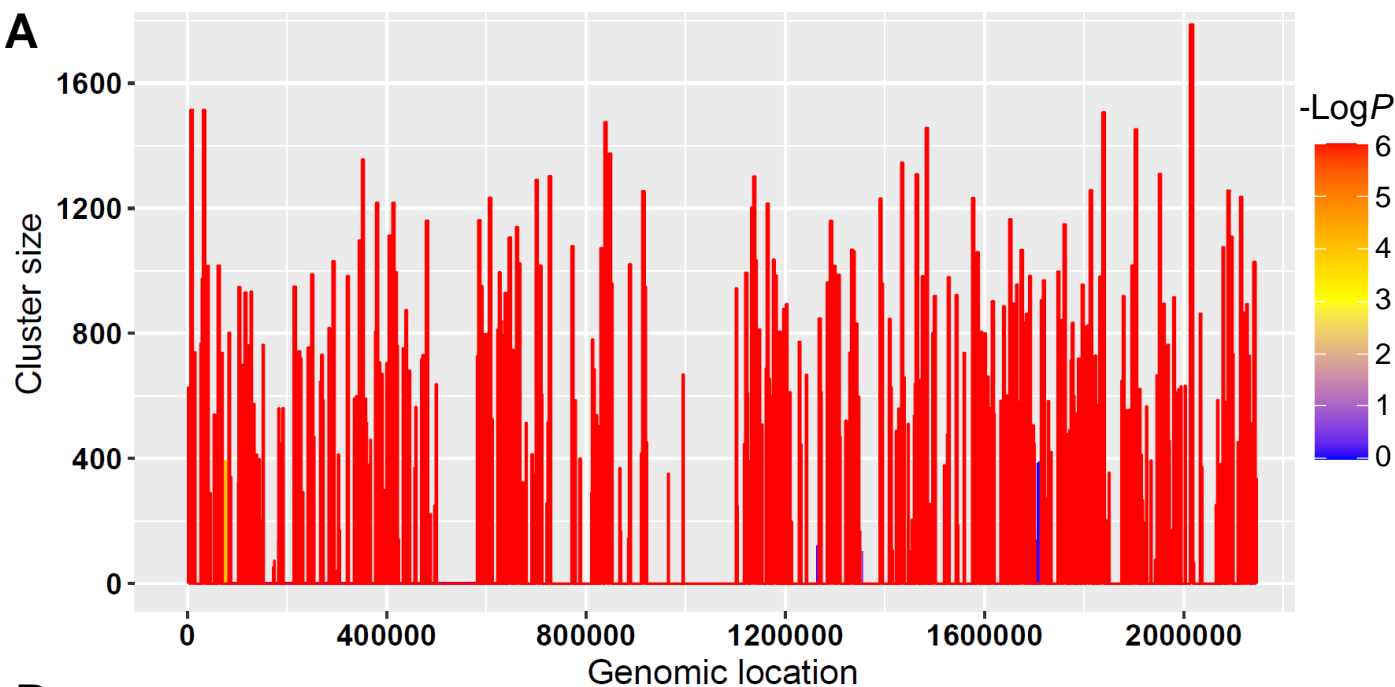

**B**

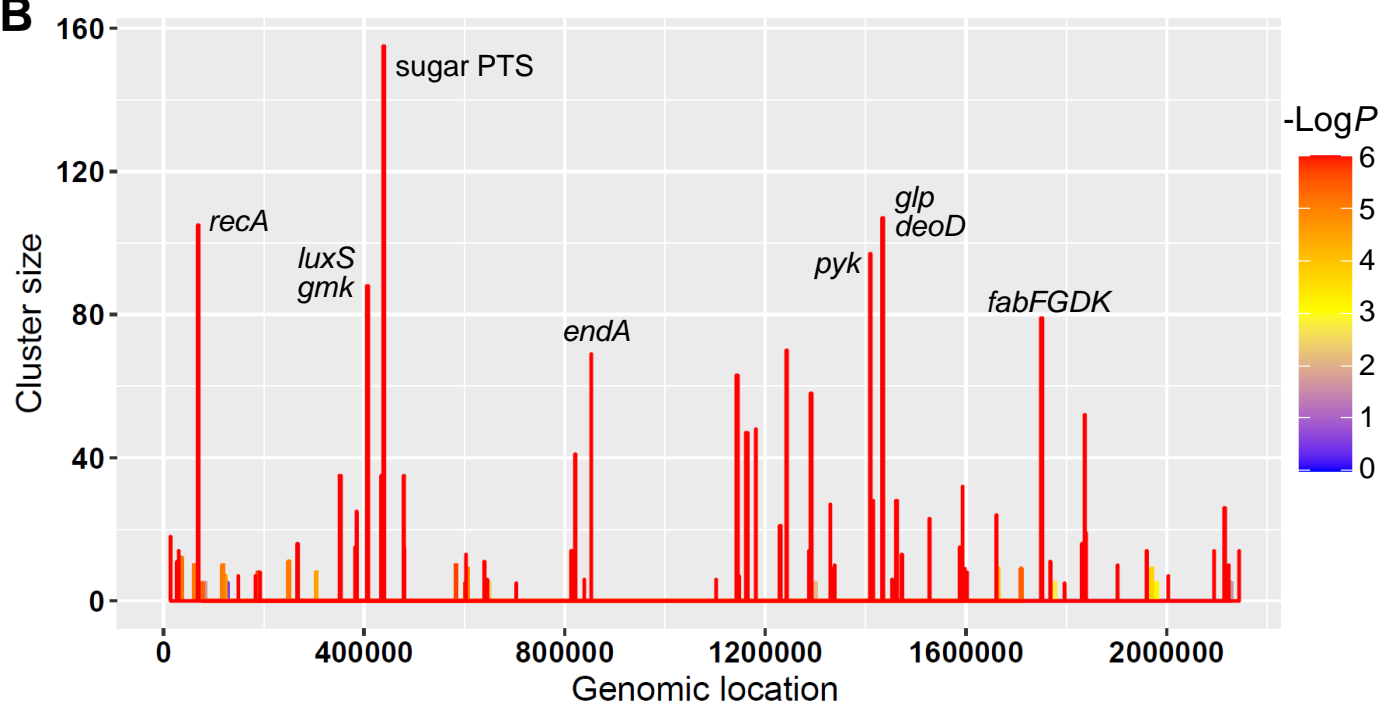

**C**

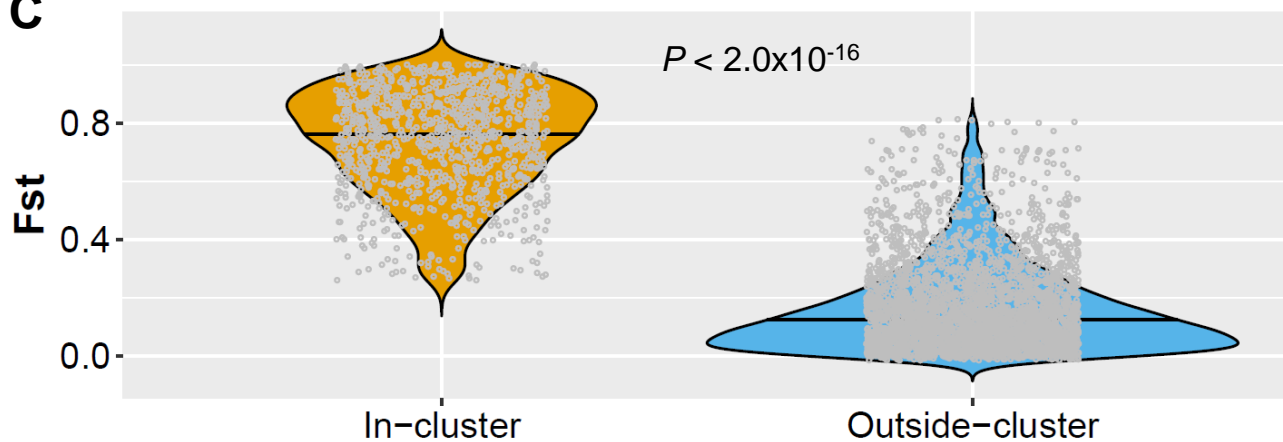

Supplement: Supplementary file 12 — Additional file 12: Fig. S4 SNP clusters with signatures of selective sweep identified by SweepCluster for S. suis genotype datasets. (A) The clusters detected from all segregating SNPs in the core genome of S. suis. (B) The clusters detected from 2,205 selected SNPs associated with population differentiation (Chi-squared test p-value ≤ 0.05). The clusters are represented as colored bars with the bar height indicating the cluster size (the number of SNPs) and the bar width indicating the spanning length. The significance of the clustering evaluated with -log10 (p-value) is indicated in gradient colors. The gene loci in the top clusters are shown. (C) Distribution comparison of population differentiation Fst between two groups of SNPs: (i) those falling into the 111 significant clusters involving 2,049 SNPs and (ii) those outside of clusters among the whole-genome core SNPs. The distribution of Fst for the latter group was constructed using a random sampling of a ninth of the 234,655 core SNPs outside clusters and the calculation of Fst was based on a window size of 10. [file 12859_2021_4533_MOESM12_ESM.pdf]

Fig. S5

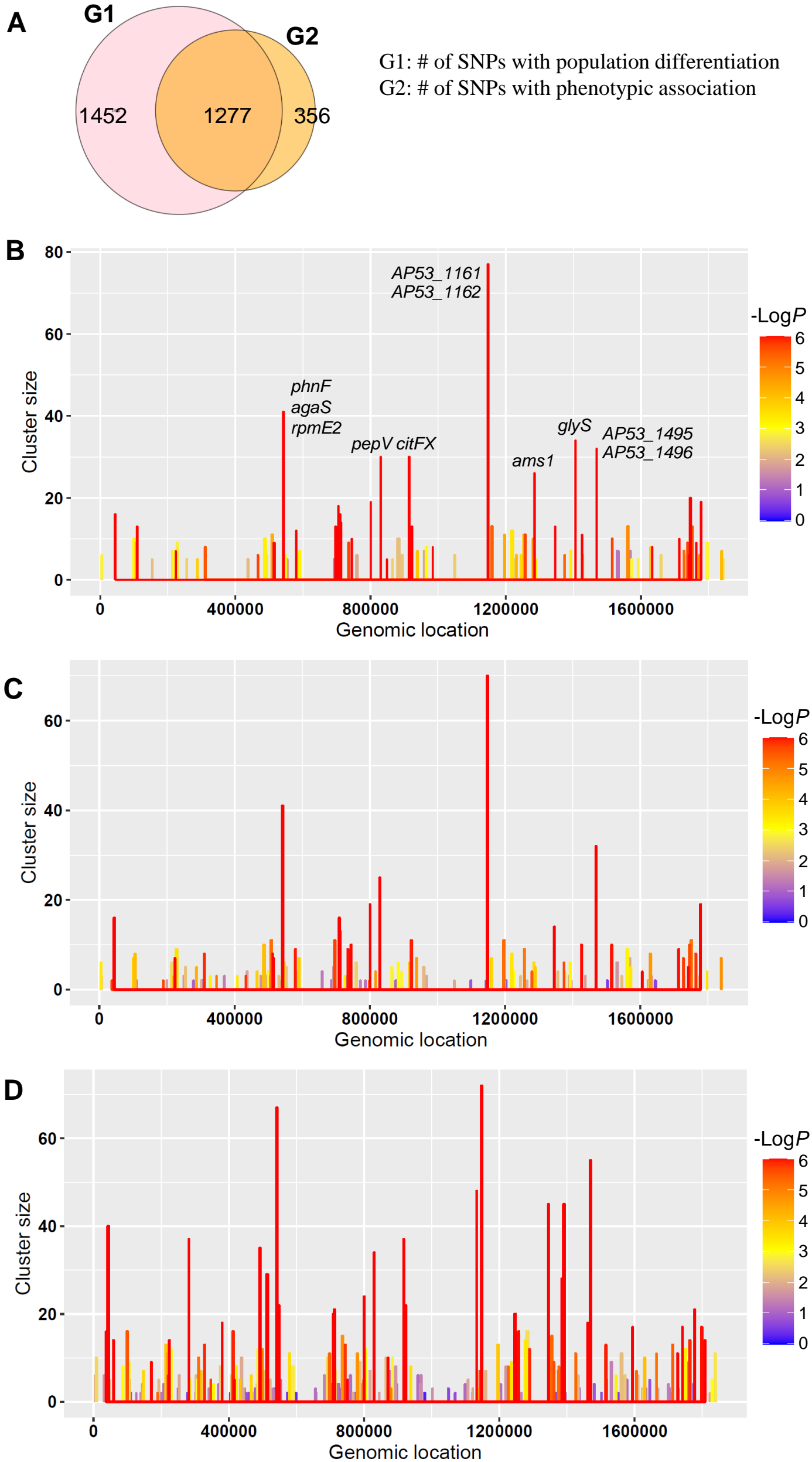

Supplement: Supplementary file 13 — Additional file 13: Fig. S5 SNP clusters with signatures of selective sweep identified by SweepCluster for S. pyogenes genotypes pre-selected using population differentiation Fst. (A) Comparison of the number of SNPs selected by population differentiation (G1, Fst ≥ 0.6) to that selected by phenotypic association (G2, Chi-squared test p-value ≤ 0.001). (B) The clusters detected from 1,631 selected SNPs with phenotypic association (the same as Fig. S3B, it is presented here for convenient comaprision). (C) The clusters detected from 1,277 SNPs selected by both methods. (D) The clusters detected from 2,729 SNPs selected by population differentiation. The clusters are represented as colored bars with the bar height indicating the cluster size (the number of SNPs in the clusters) and the bar width indicating the spanning length. The significance of the clustering evaluated with -log10 (p-value) is indicated in gradient colors. [file 12859_2021_4533_MOESM13_ESM.pdf]
