# Supplementary material for: SweepCluster: A SNP clustering tool for detecting gene-specific sweeps in prokaryotes
Source: BMC Bioinformatics. 2022 Jan 6;23:19. doi: 10.1186/s12859-021-04533-6 (PMC8734265; doi:10.1186/s12859-021-04533-6)
Supplement: Supplementary file 9 — Additional file 9: Fig. S1 The phylogenetic trees for selection of non-redundant strains. (A) The trees for each of the seven groups of strains. The grouping was based on the submission institutions. The strains selected for downstream analysis in each group are indicated in red square. (B) The final phylogenetic tree for 209 selected non-redundant genomes. (C) The two subpopulations used for identification of SNPs associated with population differentiation are indicated with numbers. [file 12859_2021_4533_MOESM9_ESM.pdf]

Fig.S1

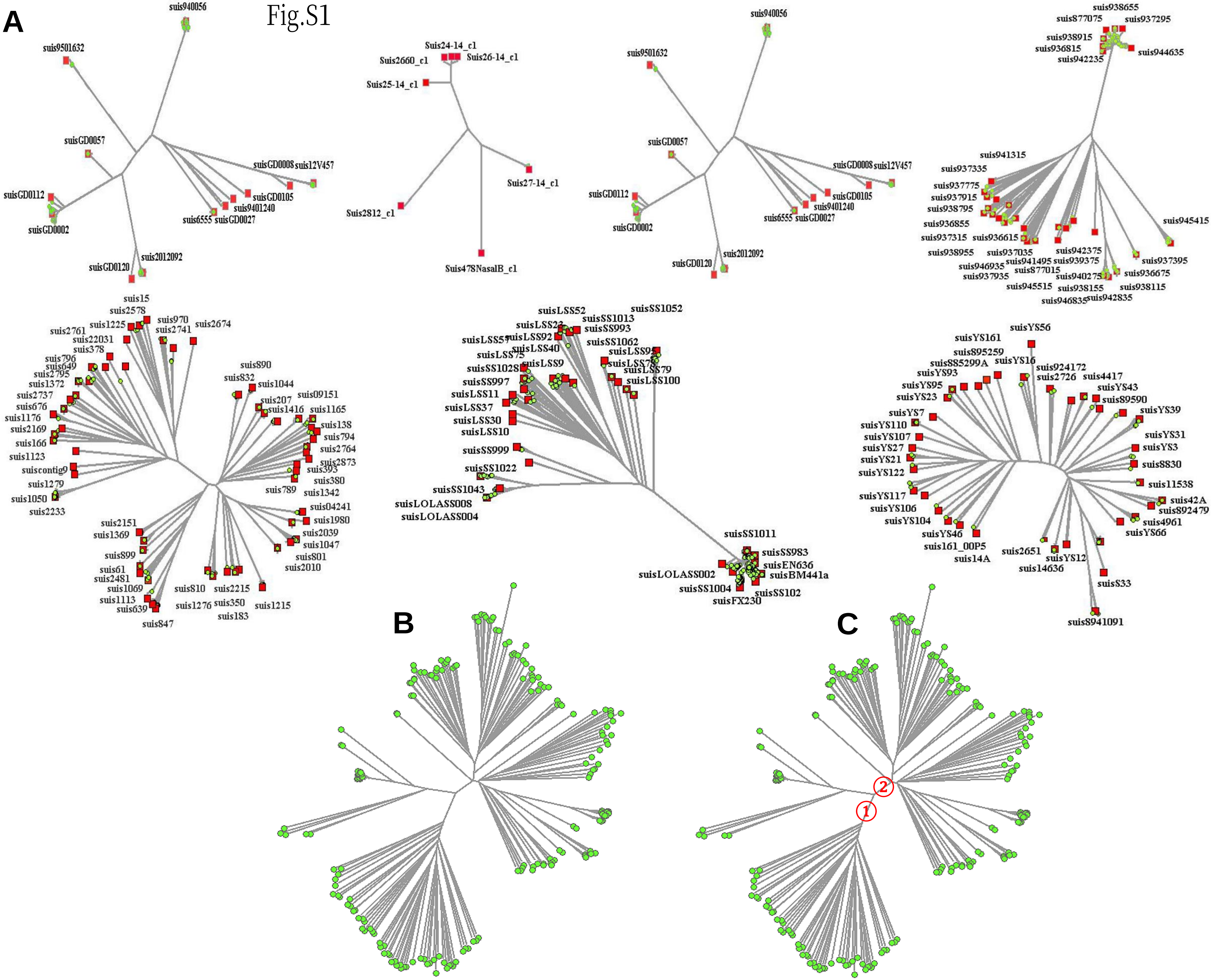
A
suis940056
suis9501632
suisGD0057
suisGD0008
suis12V457
suisGD0112
suisGD0105
suis9401240
suis6555
suisGD0027
suisGD0002
suisGD0120
suis2012092
Suis24-14_c1
Suis2660_c1
Suis26-14_c1
Suis25-14_c1
Suis27-14_c1
Suis2812_c1
Suis478NasalB_c1
suis940056
suis9501632
suisGD0057
suisGD0008
suis12V457
suisGD0112
suisGD0105
suis9401240
suis6555
suisGD0027
suisGD0002
suisGD0120
suis2012092
suis938655
suis877075
suis937295
suis938915
suis936815
suis944635
suis942235
suis941315
suis937335
suis937775
suis937915
suis938795
suis936855
suis937315
suis936615
suis938955
suis937035
suis942375
suis946935
suis941495
suis939375
suis937935
suis877015
suis940275
suis945515
suis938155
suis946835
suis942835
suis945415
suis937395
suis936675
suis938115
suis15
suis2578
suis2761
suis1225
suis970
suis2741
suis2674
suis22031
suis378
suis796
suis649
suis2795
suis1372
suis2737
suis676
suis1176
suis2169
suis166
suis1123
suiscontig9
suis1279
suis1050
suis2233
suis890
suis832
suis1044
suis09151
suis207
suis1416
suis1165
suis138
suis794
suis2764
suis2873
suis393
suis380
suis789
suis1342
suis04241
suis1980
suis2039
suis1047
suis801
suis2010
suis2151
suis1369
suis899
suis61
suis2481
suis1069
suis1113
suis639
suis847
suis810
suis2215
suis1276
suis350
suis183
suis1215
suisLSS52
suisSS1052
suisSS1013
suisLSS22
suisSS993
suisLSS57
suisLSS92
suisSS1062
suisLSS40
suisLSS95
suisLSS75
suisLSS78
suisSS1028
suisLSS9
suisLSS79
suisSS997
suisLSS100
suisLSS11
suisLSS37
suisLSS30
suisLSS10
suisSS999
suisSS1022
suisSS1043
suisLOLASS008
suisLOLASS004
suisSS1011
suisSS983
suisEN636
suisLOLASS002
suisBM441a
suisSS1004
suisSS102
suisFX230
suisYS56
suisYS161
suis895259
suis885299A
suisYS16
suisYS93
suis924172
suis2726
suis4417
suisYS95
suisYS43
suisYS23
suis89590
suisYS7
suisYS39
suisYS110
suisYS107
suisYS31
suisYS27
suisYS3
suisYS21
suis8830
suisYS122
suis11538
suisYS117
suis42A
suisYS106
suis892479
suisYS104
suis4961
suisYS46
suisYS66
suis161_00P5
suis2651
suisYS12
suis14A
suis14636
suisS33
suis8941091
B
C
1
2
